# Clinicopathological and genomic features in patients with head and neck neuroendocrine carcinoma

**DOI:** 10.1038/s41379-021-00869-9

**Published:** 2021-07-10

**Authors:** Akihiro Ohmoto, Yukiko Sato, Reimi Asaka, Naoki Fukuda, Xiaofei Wang, Tetsuya Urasaki, Naomi Hayashi, Yasuyoshi Sato, Kenji Nakano, Mayu Yunokawa, Makiko Ono, Junichi Tomomatsu, Takashi Toshiyasu, Hiroki Mitani, Kengo Takeuchi, Seiichi Mori, Shunji Takahashi

**Affiliations:** 1 Division of Medical Oncology, Cancer Institute Hospital of Japanese Foundation for Cancer Research, Tokyo, Japan; 2 Division of Pathology, Cancer Institute Hospital of Japanese Foundation for Cancer Research, Tokyo, Japan; 3 Pathology Project for Molecular Targets, Cancer Institute Hospital of Japanese Foundation for Cancer Research, Tokyo, Japan; 4 Division of Radiation Oncology, Cancer Institute Hospital of Japanese Foundation for Cancer Research, Tokyo, Japan; 5 Division of Head and Neck Oncology, Cancer Institute Hospital of Japanese Foundation for Cancer Research, Tokyo, Japan; 6 Division of Pathology, Cancer Institute, Japanese Foundation for Cancer Research, Tokyo, Japan; 7 Project for Development of Innovative Research, Cancer Precision Medicine Center, Japanese Foundation for Cancer Research, Tokyo, Japan

**Keywords:** Cancer genomics, Neuroendocrine cancer

## Abstract

Neuroendocrine carcinoma (NEC) of the head and neck is a rare type of malignancy, accounting for only 0.3% of all head and neck cancers, and its clinicopathological and genomic features have not been fully characterized. We conducted a retrospective analysis of 27 patients with poorly differentiated NEC of the head and neck seen at our institution over a period of 15 years. Patient characteristics, adopted therapies, and clinical outcomes were reviewed based on the medical records. Pathological analysis and targeted sequencing of 523 cancer-related genes were performed using evaluable biopsied/resected specimens based on the clinical data. The most common tumor locations were the paranasal sinus (33%) and the oropharynx (19%). Eighty-one percent of the patients had locally advanced disease. The 3-year overall survival rates in all patients and in the 17 patients with locally advanced disease who received multimodal curative treatments were 39% and 53%, respectively. Histologically, large cell neuroendocrine carcinoma was the predominant subtype (58% of evaluable cases), and the Ki-67 labeling index ranged from 59 to 99% (median: 85%). Next-generation sequencing in 14 patients identified pathogenic/likely pathogenic variants in *TP53*, *RB1*, PIK3CA-related genes (*PREX2*, *PIK3CA*, and *PTEN*), *NOTCH1*, and *SMARCA4* in six (43%), three (21%), two (14%), two (14%), and one (7%) patients, respectively. Sequencing also detected the *FGFR3*-*TACC3* fusion gene in one patient. The median value of the total mutational burden (TMB) was 7.1/Mb, and three patients had TMB ≥ 10. Regardless of the aggressive pathological features, our data revealed favorable clinical characteristics in the patients with locally advanced disease who received curative treatment. The lower *TP53* and *RB1* mutation prevalence rates compared to those described for small cell lung cancer suggests the biological heterogeneity of NEC in different parts of the body. Furthermore, the *FGFR3*-*TACC3* fusion gene and mutations in genes encoding the components of the NOTCH and PI3K/AKT/mTOR pathways found in our study may be promising targets for NEC of the head and neck.

## Introduction

Poorly differentiated neuroendocrine carcinoma (NEC) of the head and neck is a rare malignancy. Extrapulmonary NEC is histologically divided into pure small cell carcinoma (SCC), pure large cell neuroendocrine carcinoma (LCNEC), and combined/mixed NEC with non-neuroendocrine neoplasms [1]. According to the analysis based on the National Cancer database in the United States, SCC accounted for 0.3% of ~350 000 patients with head and neck cancer [2]. Another study that included ~160 000 patients with poorly differentiated NEC from the Surveillance, Epidemiology, and End Results (SEER) database showed that the percentage of cases derived from the oral cavity and pharynx was 0.3% of all NEC cases throughout the body [3]. Regarding the prognosis, Pointer et al. [2] showed a 2-year overall survival (OS) rate of 45% in an SCC cohort, and Wakasaki et al. [4] reviewed 21 patients with SCC of the head and neck and reported 1- and 3-year OS rates of 56% and 37%, respectively. In that study, 19% of the patients had metastatic disease at initial treatment, whereas 62% of the patients developed the metastatic disease within 3 years. These outcomes warrant further clinical investigations.

The World Health Organization (WHO) 2017 Classification of Head and Neck Tumors divides neuroendocrine neoplasms (NENs) into well-differentiated, moderately differentiated, or poorly differentiated entities (SCC and LCNEC), depending on the extent of tumor differentiation [5]. Kao et al. [6] analyzed clinicopathological features in 23 patients with head and neck NENs and revealed that poorly differentiated cases (SCC or LCNEC) had adverse characteristics as compared with well-/moderately differentiated cases (typical carcinoid or atypical carcinoid) (17% vs. 88% for the 5-year OS rate). Moreover, immunohistochemical analysis showed that the proportion of p53 overexpression and the Ki-67-labeling index were significantly higher in poorly differentiated NENs.

The treatment strategies for extrapulmonary NEC generally follow those for small cell lung cancer (SCLC), and no personalized approach dependent on the primary organ has been established. To develop novel therapies for this rare malignancy, it is critically necessary to establish its biological features. Available next-generation sequencing data for NEC of the head and neck are extremely limited, and the investigation for sinonasal NEC is centered [7]. Dogan et al. [8] conducted genomic analysis using Memorial Sloan Kettering-Integrated Mutation Profiling of Actionable Cancer Targets (MSK-IMPACT), and reported that *ARID1A* mutations were identified in all three sinonasal SCC cases, whereas all five sinonasal LCNEC cases harbored *IDH2* mutations. Here, we performed the first comprehensive analysis of clinicopathological and genomic features of this rare tumor by using targeted sequencing for 523 cancer-related genes.

## Subjects and methods

### Study overview and clinical data collection

We retrospectively reviewed the Cancer Institute Hospital of the Japanese Foundation for Cancer Research database of patients with pathologically proven poorly differentiated NEC of the head and neck for a period of 15 years (from 2005 to 2019). Along with specific morphological features, all cases were positive for at least one neuroendocrine marker (chromogranin A, synaptophysin, or CD56). The reviewed clinical data included sex, age, smoking and alcohol consumption history, past history of any cancer, primary organ, clinical stage, and treatment approach, including chemotherapy regimen. For patients with locally advanced disease, the following approaches aimed at disease cure were described as curative treatments: combined surgery, radiotherapy, and chemotherapy; combined radiotherapy and chemotherapy; combined surgery and radiotherapy; combined surgery and chemotherapy; and surgery alone. Chemotherapy alone and palliative care were described as non-curative treatments. OS and relapse-free survival (RFS) were calculated as the intervals from the initial diagnosis, where RFS was applicable to locally advanced cases that received curative treatment. In addition, pathological and genomic analyses were conducted for evaluable biopsied or resected specimens. This study was reviewed and approved by the Institutional Review Board of the Japanese Foundation for Cancer Research and conducted in accordance with the guidelines established by the Helsinki Declaration.

### Pathological review of NEC specimens

Resected or biopsied tumor tissues were formalin-fixed and paraffin-embedded, and paraffin blocks were sectioned at 5-μm thickness for hematoxylin and eosin staining and subsequent immunohistochemistry. The morphological subtype (SCC or LCNEC) and the Ki-67 labeling index were assessed for each specimen. According to the 2015 WHO Classification of Tumors of the Lung, each sample was morphologically classified as SCC or LCNEC [9]. In SCLC, tumor cells have ill-defined cell borders, scant cytoplasm, finely granular nuclear chromatin, and inconspicuous nucleoli, and the size is less than three times that of normal lymphocytes. In LCNEC, palisading or rosette-like structures are characteristically observed, and tumor cells are more than three times larger than normal lymphocytes along with ample cytoplasm and prominent nucleoli. The Ki-67-labeling index was expressed as the average percentage of positive cells in three randomly selected regions. Immunohistochemical staining for p53 and Rb was performed using all available specimens, and SMARCA4 immunostaining was performed for the *SMARCA4*-mutated specimen. When more than 60% of tumor cells were positive for p53, they were pathologically classified as “p53 overexpression”. Primary monoclonal antibodies included the following: Ki-67 (1:200; M724001-2, clone MIB-1; Agilent Technologies, Santa Clara, CA, USA), p53 (1:200; M700101-2, clone DO-7; Agilent Technologies), Rb (1:100; MA1-34070, Clone 1F8; Invitrogen, Carlsbad, CA, USA), SMARCA4/BRG1 (1:100; ab110641, clone EPNCIR111A; Abcam, Cambridge, UK), MCPyV large T-antigen (1:50; sc-136172, clone CM2B4; Santa Cruz Biotechnology, Dallas, TX, USA), CK20 (1:100; 65126, clone IT-Ks20.8; Progen, Heidelberg, Germany), and insulinoma-associated protein 1 (INSM1; 1:300; sc-271408, clone A-8; Santa Cruz Biotechnology). A CINtec p16 histology kit (Roche, Basel, Switzerland) was used for p16 immunostaining.

### Next-generation sequencing of 523 cancer-related genes

The block sectioning for genomic analysis was performed at our institution, whereas DNA/RNA extraction and targeted capture sequencing were performed by RIKEN Genesis Co., Ltd. (Tokyo, Japan). Ten slides with 5-μm thick sections of the paraffin-embedded blocks were prepared per each case. DNA and RNA samples were extracted from specimens with tumor cell proportions of more than 20% by using Maxwell RSC DNA and RNA FFPE kits (Promega, Madison, WI, USA), and the required DNA and RNA input was 40 ng each. Custom targeted capture and library preparation were conducted using a TruSight Oncology 500 Library Preparation Kit (Illumina, San Diego, CA, USA). Targeted-capture sequencing of 523 cancer-related genes was performed using the NextSeq System (Illumina) (Supplementary Table 1). In the bioinformatics analysis, TruSight Oncology 500 Local App (DNA) and TruSight Tumor 170 Local App (RNA) were used to map the reference sequence and to search for variants. In the DNA analysis, single nucleotide variants, insertions/deletions, copy number variations, total mutational burden (TMB), and microsatellite instability (MSI) were calculated [10]. In RNA analysis, gene fusion and splice variants were detected. TMB was calculated by dividing the total number of somatic single nucleotide variants and insertions/deletions by the length of the captured region. TMB-high was defined as ≥10 mutations/megabase (mut/Mb). MSI quantitative score was calculated by interrogating 130 homopolymer MSI marker sites and defined as the proportion of MSI unstable sites to the total assessed MSI sites.

Pathogenicity of each variant was interpreted using the public databases COSMIC (Catalog of Somatic Mutations in Cancer; https://cancer.sanger.ac.uk/cosmic) and ClinVar (https://www.ncbi.nlm.nih.gov/clinvar/). Briefly, COSMIC was used to verify the registered data about somatic variants, and ClinVar was used for categorizing the clinical significance of the germline variants. Non-synonymous variants not found in these databases were classified based on the predicted effect on the protein product. Nonsense variants and variants changing the canonical splice sites (i.e., ±2 base pairs), as well as frameshift insertions and deletions, were judged as deleterious unless they occurred in the last exon. To predict whether an amino acid substitution affected protein function, SIFT (http://sift.jcvi.org) and PolyPhen-2 (http://genetics.bwh.harvard.edu/pph2/) were used. According to the above algorithm and a literature review, each variant was comprehensively classified as deleterious, benign, or variant of uncertain significance. The Integrative Genomics Viewer was used for the inspection and validation of the respective variants [11].

### Validation of the *FGFR3-TACC3* gene fusion by nested RT-PCR

Nested reverse transcriptase-polymerase chain reaction (RT-PCR) for the *FGFR3*-*TACC3* gene fusion was conducted using extracted RNA. For the 1st PCR, two primers with different sequences were used. The primer sequences used for PCR were as follows: (1st PCR#1) *FGFR3* forward: 5′-CATGATCATGCGGGAGTGCTG-3′; *TACC3* reverse, 5′-AGTTCCAGGTTCTTCCCGTGGAG-3′; (1st PCR#2) *FGFR3* forward: 5′-CACACACGACCTGTACATGATCATGC-3′; *TACC3* reverse: 5′-CCATGATCTTCCCCAGTTCCAGG-3′; (2nd PCR) *FGFR3* forward: 5′-ACCTTCAAGCAGCTGGTGGAG-3′; *TACC3* reverse: 5′-GTTCTTCCCGTGGAGCTCCTC-3′.

### Detection of human papillomavirus (HPV) by RT-PCR

p16 immunopositivity was validated using nested RT-PCR for HPV types 16 and 18. The primer sequences were as follows: (1st PCR, HPV type 16) forward, 5′-GCGACGTGAGGTATATGACT-3′ and reverse, 5′-GGTTTCTCTACGTGTTCTTG-3′; (1st PCR, HPV type 18) forward, 5′-TATACCGCATGCTGCATGCC-3′ and reverse, 5′-ACGGTTTCTGGCACCGCAGG-3′; (2nd PCR, HPV type 16) forward, 5′-ATTAGTGAGTATAGACATTA-3′ and reverse, 5′-GGCTTTTGACAGTTAATACA-3′; (2nd PCR, HPV type 18) forward, 5′-ATTAGAGAATTAAGACATTA-3′ and reverse, 5′-GGTTTCTGGCACCGCAGGCA-3′.

### Detection of HPV by in situ hybridization (ISH)

ISH was conducted using a Wide Spectrum HPV biotinylated DNA probe (Y1404; DAKO, Troy, MI, USA) for HPV types 6, 11, 16, 18, 31, 33, 35, 45, 51, and 52. Briefly, deparaffinization and rehydrated specimens were treated with proteinase and immersed in 0.3% H_2_O_2_ in methanol for 20 min. After dehydration and immersion, the probe and target DNA were incubated at 95 °C for 5 min prior to overnight hybridization at 37 °C. Detection of the hybridized probe was performed using the Dako GenPoint tyramide signal amplification system for biotinylated probes (DAKO) with the application of a primary streptavidin–peroxidase conjugate (1:400 dilution) and secondary streptavidin peroxidase. The slides were counterstained with hematoxylin, and punctate nuclear staining (brown nuclear dots) in tumor cells was judged as ISH positive.

### Statistical analysis

Differences in categorical variables between groups were analyzed using the Fisher’s exact test. Survival curves were estimated using the Kaplan–Meier method, and *P-*values were calculated using the log-rank test. Univariate and multivariate analyses of risk factors for OS and RFS were performed using the Cox proportional hazards regression model. For multivariate analysis, clinically valid factors with low *P*-values according to univariate analysis were included. Effects were considered statistically significant at a two-sided *P* < 0.05. All statistical analyses were performed using EZR (v.1.4.1; Saitama Medical Center, Jichi Medical University, Shimotsuke, Japan), which is based on R and R commander (http://www.jichi.ac.jp/saitama-sct/SaitamaHP.files/download.html) [12].

## Results

### Clinical features in patients with poorly differentiated NEC

The clinical data of the 27 patients included in this study are summarized in Table 1. The median patient age was 64 years, and the clinical stage at diagnosis based on the American Joint Committee on Cancer staging system was locally advanced disease (stage III–IVB) in 22 patients (85%) and metastatic disease (stage IVC) in four patients (15%). The common tumor location was the paranasal sinus in nine patients (33%), oropharynx in five patients (19%), nasal cavity in four patients (15%), salivary gland in three patients (11%), and hypopharynx in three patients (11%). Six of 24 patients (25%) had a history of some cancer (three patients with colorectal cancer, one patient with prostate cancer, one patient with esophageal cancer, and one patient with breast cancer). Among the aforementioned six patients, one was concurrently diagnosed with colorectal cancer and NEC of the oropharynx. The percentage of smokers was 79%, and the median Brinkman Index in the available 24 cases was 400 (range, 0–1480). Information regarding the initial treatment was available for 24 patients. Six patients with locally advanced disease received radiotherapy/chemotherapy, four patients received surgery/radiotherapy/chemotherapy, four patients underwent surgery, and three patients received chemotherapy. In total, 17 patients (77%) received at least one treatment aimed at disease cure. Two patients with the metastatic disease received chemotherapy (cisplatin/irinotecan regimen).

**Table 1:** Clinical features in 27 patients with neuroendocrine carcinoma of the head and neck.

Variables	Number (%)
Age	Median 64 (range, 39–88)
Gender	
Male	20 (74%)
Female	7 (26%)
Smoking	
Yes	19/24 (79%)
Alcohol	
Yes	15/24 (63%)
Past history of any cancer	6/24 (25%)
Tumor location	
Paranasal	9 (33%)
Oropharynx	5 (19%)
Nasal	4 (15%)
Salivary	3 (11%)
Hypopharynx	3 (11%)
Oral cavity	1 (4%)
Larynx	2 (7%)
Clinical stage (AJCC stage)	
III	4 (15%)
IVA	14 (52%)
IVB	4 (15%)
IVC	4 (15%)
NA	1 (4%)
Initial treatment	
(Locally advanced disease)	
OP/RT/CT	4 (15%)
OP/RT	2 (7%)
OP/CT	1 (4%)
RT/CT	6 (22%)
OP	4 (15%)
CT	3 (11%)
BSC	2 (7%)
(Metastatic disease)	
CT	2 (7%)
NA	2 (7%)

*OP* operation, *RT* radiation therapy, *CT* chemotherapy, *BSC* best supportive care, *NA* not available.

**Fig. 1:**
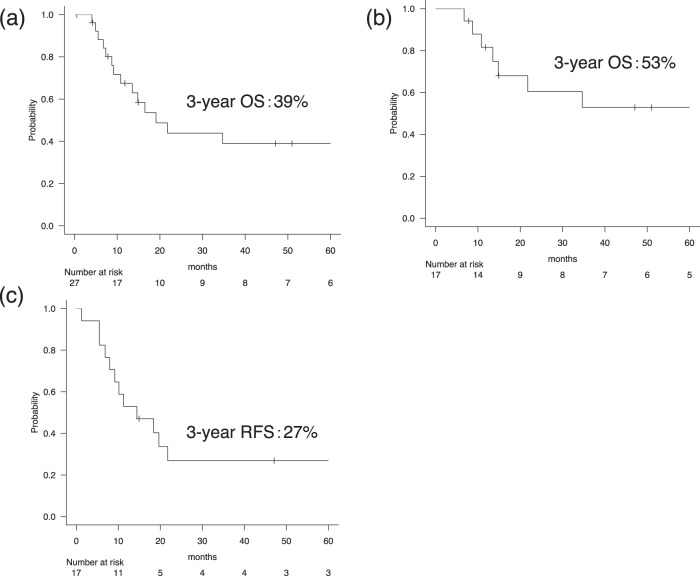
Overall survival (OS) curves in patients with head and neck neuroendocrine carcinoma. OS in (**a**) all 27 patients and (**b**) in the 17 patients with locally advanced disease who received multimodal curative treatments. **c** Relapse-free survival (RFS) rate in the 17 patients with locally advanced disease who received multimodal curative treatments.

Three-year OS rates in the entire cohort and in the 17 patients with locally advanced disease receiving curative treatments were 39% and 53%, respectively (Fig. 1a, b). The 1-year OS rate in the four metastatic cases was 50%, and all patients died within 2 years after the initial diagnosis. Univariate analysis for OS in the entire cohort identified the only metastatic disease as a significant factor for poor OS [hazard ratio (HR) = 3.5 (1.1–11.8), *P* = 0.04]. Age (<65 years vs. ≥65 years) and pathological subtype (SCC vs. LCNEC) tended to be significant (3-year OS rate: 24% vs. 60%, *P* = 0.12; 3-year OS rate: 25% vs. 58%, *P* = 0.12) and were incorporated into the multivariate model along with the metastatic disease. Multivariate analysis confirmed metastatic disease as the only factor [HR 5.1 (1.3–19.0), *P* = 0.02]. Other factors [sex, smoking history, alcohol consumption history, past history of any cancer, and tumor location (nasal/paranasal vs. others] and the Ki-67 index in tumor specimens (Ki-67 index ≥90% vs. <90%) were not significantly associated with OS. For patients with locally advanced disease that received curative treatment, 12 (71%) experienced clinical relapse, and the 3-year RFS rate was 27% (Fig. 1c). The second-line treatment for relapsed cases with an initial curative treatment was radiotherapy in six patients, surgery in two patients, surgery/radiotherapy/chemotherapy in one patient, surgery/chemotherapy in one patient, and chemotherapy in one patient. Another patient received palliative care without active treatment.

### Morphological features and immunostaining

**Fig. 2:**
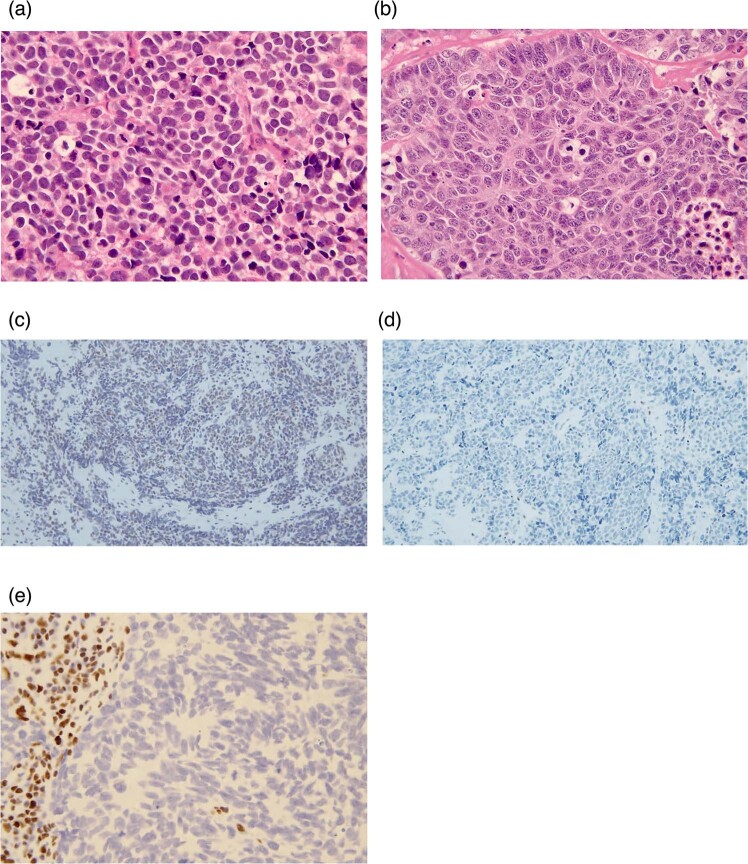
Morphological subtypes of poorly differentiated neuroendocrine carcinoma (original magnification, ×400). **a**, **b** Small cell carcinoma (**a**, SCC) and large cell neuroendocrine carcinoma (**b**, LCNEC). **c**, **d** Rb immunostaining for resected/biopsied specimens. Representative images of an Rb-positive specimen (**c**) and an Rb-deficient specimen (**d**) are shown (Rb antibody dilution, 1:100; original magnification, ×200). **e** Immunostaining for SMARCA4/BRG1 in the specimen harboring a *SMARCA4* mutation (patient ID-11). BRG1 was lost in the tumor (SMARCA4/BRG1 antibody dilution, 1:100; original magnification, ×400).

Pathologic analysis data for the evaluable six resected and 18 biopsied specimens are presented in Table 2. All patients were pathologically diagnosed with poorly differentiated NEC. The morphological subtype was SCC in 10 cases (42%) and LCNEC in 14 cases (58%) (Fig. 2a, b). The anatomical site in 10 tumors with SCC was nasal cavity in five cases, hypopharynx in two cases, paranasal sinus in one case, larynx in one case, and oropharynx in one case, respectively. A small mixture of squamous cell carcinoma components was observed in two specimens (ID-6 and ID-22). The Ki-67 labeling index in the 14 evaluable samples ranged from 59% to 99% (median: 85%), where the median Ki-67 index in three series of SCC and 11 series of LCNEC was 63% (range, 59–81%) and 87% (range, 60–99%), respectively. Immunostaining of evaluable specimens showed p53 overexpression in 18 of 19 (95%) evaluable cases and Rb loss in six of 16 cases (38%) (Fig. 2c, d). As described in the next section, one specimen with a *SMARCA4* mutation (ID-11) exhibited BRG1 loss (Fig. 2e). This case was located in the sinonasal tract and morphologically categorized as LCNEC. Regarding neuroendocrine markers, four of 24 evaluable specimens exhibited CD56-positivity without chromogranin A and synaptophysin expression (ID-7, -18, -20, and -24). No significant difference was detected in OS between these four cases and 20 cases with chromogranin A or synaptophysin expression (*P* = 0.35). For the four cases, we conducted immunostaining of INSM1 as a highly sensitive and specific neuroendocrine marker for SCLC [13]. Only two cases were INSM1-positive (ID-18 and -24), and two cases without INSM1 exhibited morphological features consistent with SCC and LCNEC.

**Table 2:** Pathological analysis for 24 resected/biopsied specimens.

Sample ID	Available tumor specimen	Tumor location	Pathological subtype	Mixture of squamous cell carcinoma component	Ki-67 index	p16 expression	Rb expression	p53 overexpression	*RB1* mutation	*TP53* mutation
1	Biopsy	Paranasal sinus	SCC	No	NA	NA	NA	Pos	Not analyzed	Not analyzed
2	Biopsy	Oral	LCNEC	No	66%	NA	Pos	Pos	Neg	Neg
3	Biopsy	Nasal cavity	SCC	No	81%	NA	Neg	NA	Pos	Pos
4	Biopsy	Oropharynx	LCNEC	No	85%	Pos	Neg	Pos	Pos	Neg
5	Biopsy	Nasal cavity	SCC	No	NA	NA	NA	Pos	Not analyzed	Not analyzed
6	Biopsy	Paranasal sinus	LCNEC	Yes	91%	NA	Neg	Pos	Neg	Neg
7	Resection	Paranasal sinus	LCNEC	No	NA	NA	NA	Neg	Not analyzed	Not analyzed
8	Biopsy	Paranasal sinus	LCNEC	No	99%	NA	Neg	Pos	Neg	Pos
9	Biopsy	Larynx	SCC	No	NA	NA	NA	NA	Not analyzed	Not analyzed
10	Biopsy	Hypopharynx	SCC	No	NA	NA	NA	NA	Not analyzed	Not analyzed
11	Biopsy	Paranasal sinus	LCNEC	No	72%	NA	Pos	Pos	Neg	Neg
12	Biopsy	Hypopharynx	SCC	No	NA	NA	NA	NA	Not analyzed	Not analyzed
13	Biopsy	Paranasal sinus	LCNEC	No	84%	NA	Neg	Pos	Pos	Pos
14	Biopsy	Nasal cavity	SCC	No	64%	NA	Pos	Pos	Neg	Neg
15	Resection	Salivary gland	SCC	No	59%	NA	Pos	Pos	Neg	Pos
16	Biopsy	Nasal cavity	SCC	No	NA	NA	NA	NA	Not analyzed	Not analyzed
17	Biopsy	Paranasal sinus	LCNEC	No	NA	NA	Neg	Pos	Not analyzed	Not analyzed
18	Resection	Oropharynx	LCNEC	No	87%	Neg	Pos	Pos	Neg	Neg
19	Biopsy	Oropharynx	LCNEC	No	92%	Pos	Pos	Pos	Neg	Neg
20	Biopsy	Oropharynx	LCNEC	No	93%	Neg	Pos	Pos	Neg	Neg
21	Resection	Salivary gland	LCNEC	No	NA	NA	Pos	Pos	Not analyzed	Not analyzed
22	Resection	Paranasal sinus	LCNEC	Yes	96%	NA	Pos	Pos	Neg	Pos
23	Biopsy	Oropharynx	SCC	No	NA	Neg	NA	Pos	Not analyzed	Not analyzed
24	Resection	Larynx	LCNEC	No	60%	NA	Pos	Pos	Neg	Pos

*SCC* small cell carcinoma, *LCNEC* neuroendocrine large cell carcinoma, *NA* not available, *Pos* positive, *Neg* negative.

We then performed immunostaining of p16 in five specimens from oropharynx, with two cases exhibiting strong and diffuse nuclear and cytoplasmic staining (ID-4 and -19), whereas three other cases were p16-negative. RT-PCR for HPV detected HPV type 16 in one case (ID-19), with ISH for ID-19 exhibiting a punctate nuclear-staining pattern. Moreover, Merkel cell polyomavirus (MCV) and CK20 immunostaining of two specimens from the salivary gland revealed two cases that exhibited both MCV- and CK20-negativity (ID-15 and -21), with the tumor cell size in these specimens larger relative to typical Merkel cell carcinoma.

### Gene alterations and the TMB/MSI status revealed by next-generation sequencing

As a result of a pathological specimen review, 14 poor-differentiated NEC samples in total were judged as suitable for genomic analysis. The average unique coverage depth was 318.9× (range, 157.1–525.4×). The gene variants and fusion genes detected in this analysis are summarized in Table 3. Briefly, pathogenic/likely pathogenic variants in *TP53*, *RB1*, *PIK3CA*-related genes (*PREX2*, *PIK3CA*, and *PTEN*), *NOTCH1*, and *SMARCA4* were detected in six (43%), three (21%), two (14%), two (14%), and one (7%) cases, respectively. Five out of six cases with *TP53* mutations were also pathologically classified as “p53 overexpression”, and all three cases with *RB1* mutations were negative for Rb expression (Table 2). The *FGFR3*-*TACC3* and *SEC11C*-*MYC* fusion genes were detected in patients ID-14 and ID-4, respectively. The median value of TMB was 7.1 mut/Mb (range, 3.9–17.2), and three samples had TMB ≥ 10 (one sample with TMB ≥ 17) (Table 3). The median MSI quantitative score was 0.03 (range, 0.00–0.06). The Brinkman index in the three TMB-high cases was 450, 430, and 220, respectively.

**Table 3:** Pathogenic/likely pathogenic variants detected by next-generation sequencing.

Patient ID	Gene	VAF	1000 Genomes Browser	Nucleotide change	Type of mutation	Amino acid change	COSMIC recurrence	ClinVar	SIFT	PolyPhen2	Fusion gene	TMB (mut/Mb)	MSI quantitative score	Clinical stage	OS (days)	OS status
2	*HIST3H3*	0.49		c.203_204delTCinsAA	Nonsense	p.F68*	NA	NA	NA	NA		10.3	0.000	Locally advanced	3405	Alive
	*CDKN2A, RP11-145E5.5*	0.22		c.374_384delATGTCGCACGG	Frameshift	p.D125fs		NA	NA	NA						
	*ANKRD26*	0.16		c.2084 C > G	Nonsense	p.S695*	NA	NA	NA	NA						
3	*TP53*	0.90	rs11540652	c.743 G > A	Missense	p.Arg248Gln	1205	Pathogenic	Damaging	Probably damaging		3.9	0.042	Locally advanced	329	Dead
	*RB1*	0.77		c.713_714delCA	Frameshift	p.P238fs	NA	NA	NA	NA						
4	*RB1*	0.57	rs1131690858	c.2520 + 3_2520 + 6del	Splice donor site		2	Pathogenic	NA	NA	*SEC11C*-*MYC*	6.3	0.000	Metastatic	581	Dead
6	None											7.1	0.030	Locally advanced	411	Dead
8	*TP53*	0.85		c.1027 G > T	Nonsense	p.E343*	25	NA	NA	NA		5.5	0.022	Metastatic	124	Dead
11	*SMARCA4*	0.37		c.3031delA	Frameshift	p.M1011fs	NA	NA	NA	NA		7.1	0.041	Locally advanced	3491	Alive
13	*RB1*	0.46	rs398123331	c.1399 C > T	Nonsense	p.Arg467Ter	14	Pathogenic	NA	NA		5.5	0.030	Locally advanced	2295	Alive
	*TP53*	0.88	rs876660726	c.902delC	Frameshift	p.Pro301Glnfs	NA	Pathogenic	NA	NA						
14	None										*FGFR3*-*TACC3*	4.7	0.050	Metastatic	502	Dead
15	*TP53*	0.41	rs397516436	c.637 C > T	Nonsense	p.Arg213Ter	636	Pathogenic	NA	NA		4.7	0.046	Locally advanced	205	Dead
18	None											11.0	0.030	Locally advanced	1055	Dead
19	*NOTCH1*	0.20	NA	c.4222 G > T	Nonsense	p.Glu1408Ter	1	Pathogenic	NA	NA		7.1	0.010	Locally advanced	1552	Alive
20	*PREX2*	0.11		c.3271 G > T	Nonsense	p.G1091*	NA	NA	NA	NA		17.2	0.010	Locally advanced	1433	Alive
	*NOTCH1*	0.53		c.1417 G > T	Nonsense	p.E473*	NA	NA	NA	NA						
22	*PIK3CA*	0.34	rs1057519941	c.1031 T > G	Missense	p.Val344Gly	33	Likely pathogenic	Damaging	Probably damaging		7.1	0.058	Locally advanced	238	Dead
	*PTEN*	0.55	rs121909224	c.388 C > T	Nonsense	p.Arg130Ter	145	Pathogenic	NA	NA						
	*TP53*	0.81		c.406 C > T	Nonsense	p.Q136*	75	NA	NA	NA						
24	*FAT1*	0.62		c.7259 C > G	Nonsense	p.S2420*	NA	NA	NA	NA		7.1	0.056	Locally advanced	454	Alive
	*TP53*	0.50	rs876659802	c.833 C > T	Missense	p.Pro278Leu	119	Pathogenic	Damaging	Probably damaging						

*VAF* variant allele frequency, *TMB* total mutational burden, *MSI* microsatellite instability, NA not available.

**Fig. 3:**
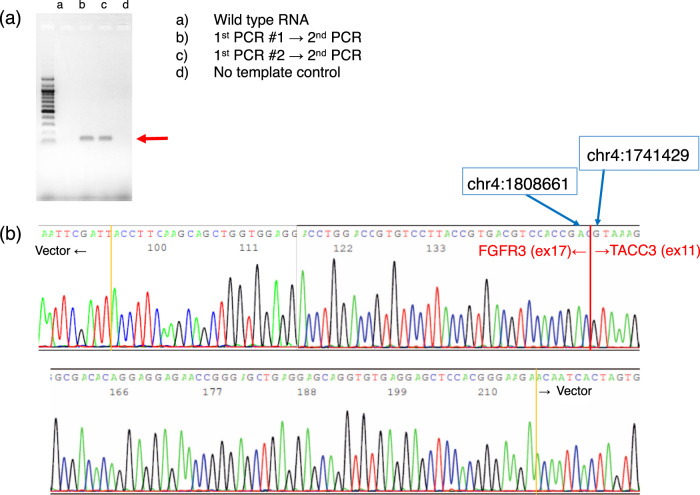
Validation of *FGFR3*-*TACC3* gene fusion by Sanger sequencing. **a** Nested RT-PCR was conducted using extracted RNA, and the available PCR product underwent Sanger sequencing. Two types of PCR products were generated after nested RT-PCR. **b** Sanger sequencing revealed gene fusion between *FGFR3* (NM_000142 exon17) and *TACC3* (NM_006342 exon11).

To validate the *FGFR3*-*TACC3* fusion gene detected by next-generation sequencing, nested RT-PCR was conducted using extracted RNA, as described in the Subjects and Methods section. Two types of PCR products were obtained using nested RT-PCR (Fig. 3a). Sanger sequencing confirmed gene fusion between *FGFR3* (NM_000142 exon 17) and *TACC3* (NM_006342 exon 11) (Fig. 3b).

**Fig. 4:**
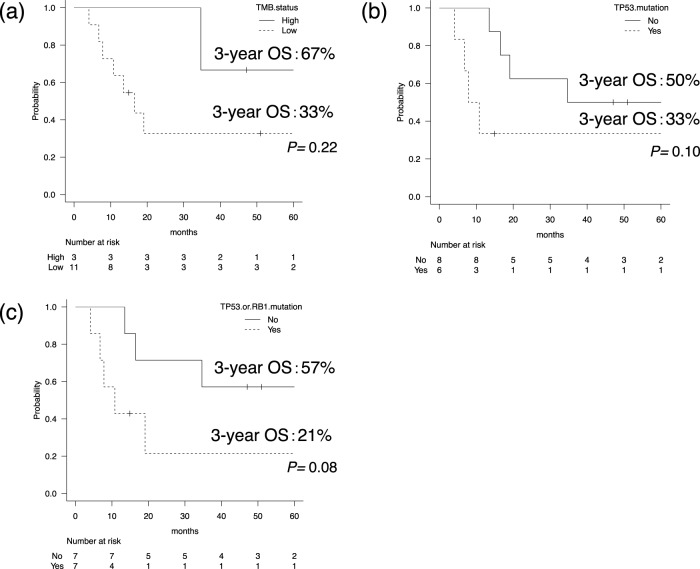
Overall survival (OS) curves in subgroups. Comparisons of the overall survival (OS) between three patients with high total mutational burden (TMB-high; ≥10 mut/Mb) and 11 cases with TMB-low (**a**) between six patients harboring a *TP53* mutation and eight without this mutation (**b**) or between seven patients harboring a *TP53* or *RB1* mutation and seven patients without these mutations (**c**).

With regard to the prognosis, there was no significant difference between the three cases with TMB-high (≥10 mut/Mb) and 11 cases with TMB-low (3-year OS 67% vs. 33%, *P* = 0.22) (Fig. 4a). Patients with *TP53* mutations or those with *TP53* or *RB1* mutations tended to have poorer OS compared with others, although the difference was not statistically significant (3-year OS: 33% vs. 50%, *P* = 0.10; 3-year OS: 21% vs. 57%, *P* = 0.08) (Fig. 4b, c).

## Discussion

This is the first comprehensive analysis of the clinicopathological and genomic features of poorly differentiated NEC of the head and neck. In terms of the prognosis, treatments aimed at disease cure with a combination of surgery, radiotherapy, or chemotherapy resulted in favorable OS in locally advanced cases, although >70% of patients experienced clinical relapse at 3 -years after the initial diagnosis. In addition, a low proportion of metastatic cases with poor OS (1-year OS rate, 50%) and a high proportion (85%) of locally advanced cases in this study might explain favorable outcomes in the entire cohort. The predominant distribution of locally advanced cases is consistent with previous studies [2, 4]. According to the National Cancer Database study of 415 patients with non-metastatic sinonasal carcinoma with neuroendocrine differentiation, including 172 with sinonasal NEC, multimodal treatments resulted in better OS as compared with unimodal treatments [14]. Results of that large-scale analysis agree with those of the present study. This clinical behavior contrasts with features of gastroenteropancreatic (GEP)-NEC as the most common extrapulmonary entity [15]. A previous study of a cohort of patients with GEP-NEC by Walter et al. [16] showed that 78% of patients harbored metastatic lesions at diagnosis. Therefore, curative strategies are unsuitable for most GEP-NEC cases, as they have metastatic disease at presentation. The present results thus highlight the unique clinical features of NEC of the head and neck.

According to pathologic analysis, our cases exhibited highly proliferative tumor cells (median Ki-67 index > 80%), which reflects aggressive pathological features. One interesting finding of this study was the discordance between favorable clinical outcomes and aggressive pathological features. Regarding the morphological distribution, we found that LCNEC was more frequent than SCC (58% vs. 42%). By contrast, SEER database analysis showed a higher proportion of SCC (65% vs. 35%) [3]. The discordance between the two studies might be related to differences in sample size or pathologic assessment method. In the present study, an expert pathologist reclassified all cases based on uniform morphological criteria, as described in the Subjects and Methods section. Regarding the specimens from salivary glands, discrimination from Merkel cell carcinoma is important. The two cases exhibited tumor cell size larger than Merkel cell carcinoma along with MCV/CK20-negativity, and we diagnosed them with SCC and LCNEC. p16 overexpression in head and neck NEC is mechanically induced by loss of both RB1 and the HPV E7 oncoprotein in tumor specimens, as Alos et al. [17] showed that 14 of 19 cases with head and neck NEC exhibited p16 overexpression, whereas HPV DNA was never detected by PCR or ISH. According to a similar analysis of HPV-related head and neck LCNEC, six of 10 specimens were p16-positive, whereas ISH detected high-risk HPV in only three cases [18]. In the present study, we considered that p16 overexpression was induced by Rb loss in patient ID-4 and by HPV type 16 in patient ID-19. Comprehensively, p16-positivity is nonspecific, and confirmation by HPV testing is required. Unlike squamous cell carcinoma from oropharynx, the favorable clinical impact of HPV in NEC remains undetermined [18].

The genomic analysis detected *TP53* and *RB1* mutations in 43% and 21% of the cases, respectively. According to large-scale whole-genome sequencing analysis for SCLC, *TP53* and *RB1* mutation-prevalence rates were 100% and 93%, respectively, and their frequencies in the present study were not as high as those in SCLC [19]. One interesting finding was the discrepant proportions between *TP53*-mutated and p53-overexpression cases and between *RB1*-mutated and Rb-loss cases. According to a previous report on poorly differentiated pancreatic NEC, the percentage of cases with p53 overexpression and Rb loss was 7/7 (100%) and 7/7 (100%), and those with *TP53* and *RB1* mutations were 4/7 (57%) and 5/7 (71%), respectively [20]. Similar to our cohort, all patients with *TP53* and *RB1* mutations in that study exhibited abnormal expression of p53 and Rb. As shown in Fig. 4, our data suggested that there might be some difference in clinical behavior between cases with SCLC-like features (i.e., those harboring a *TP53* or *RB1* mutation) and other cases.

Moreover, deleterious variants were identified in *NOTCH1*, *SMARCA4*, and genes encoding the components of the PI3K/AKT/mTOR pathway (*PREX2*, *PIK3CA*, and *PTEN*). Notch-1 signaling suppresses tumor progression in NENs [21, 22]. In the present study, we found two nonsense variants in two patients (ID-19 and -20), with these mechanistically considered inactivating mutations. Previous whole-genome analysis of SCLC samples classified *NOTCH1* as significantly damaged genes [19]. Moreover, PI3K/AKT/mTOR signaling is a major pathway implicated in the pathogenesis of well-differentiated pancreatic neuroendocrine tumors [23]. A previous comprehensive genomic analysis identified *PTEN*, *TSC1*/*TSC2*, and *PIK3CA* mutations that altered proteins involved in PI3K/AKT/mTOR signaling in 17%, 29%, and 11% of patients, respectively [24–26]. In the present study, the *PTEN* variant in patient ID-22 was a nonsense mutation, whereas the *PIK3CA* variant (p.Val344Gly) in the same patient was predicted to confer a gain of function [27]. These variants represent suitable targets for mTOR inhibitors to prevent upregulation of mTOR complex 1 [28].

SMARCA4 is a subunit of the switch/sucrose non-fermentable chromatin remodeling complex, which functionally cooperates with EZH2 to stabilize the polycomb repressive complex 2 [29, 30]. Therefore, EZH2 inhibitors are a promising option for treating switch/sucrose non-fermentable chromatin remodeling complex-deficient malignancies. Although there have been no reports on the functional role of the *SMARCA4* variant (p.M1011fs) found in patient ID-11, preclinical functional analysis might indicate the benefit of this class of agents in the future. It is debatable whether the cases of the tumors derived from ethmoid sinus should be diagnosed with NEC or SMARCA4-deficient sinonasal carcinoma [31]. The specimen from patient ID-11 exhibited a large cell morphology accompanied by upregulated expression of chromogranin A, synaptophysin, and CD56. Although this morphological and immunohistochemical feature is consistent with a 10-case series described by Agaimy et al. [31], distinction from LCNEC is practically complicated. Teratocarcinosarcoma is another possible diagnosis. In this patient, the available specimen was a biopsy sample, and we cannot completely rule out the coexistence of teratoma, carcinoma, or a sarcoma component, which is typical of teratocarcinosarcoma [32]. In addition, uniform tumor-contrast enhancement using computerized tomography and magnetic resonance imaging does not positively support the diagnosis. In our analysis, two patients had components of squamous cell carcinoma; however, owing to the low mixture ratio, the possibility of distorted genomic data is unlikely. In the present study, *ARID1A* and *IDH2* mutations highlighted in by previous studies were not detected, partly due to the small sample size [8].

Several studies have highlighted the heterogeneous molecular background of NEC, which is dependent on the primary organ. SCLC is the most common NEC subtype all over the body, and has diagnostic thresholds and risk factors similar to head and neck NEC. According to whole-genome sequencing of 110 SCLC specimens, the mutation frequency of *TP53* and *RB1* was 100% and 93%, respectively, and inactivating mutations in NOTCH family genes were detected in 25% of the cases [19]. That study also identified activating mutations in *BRAF*, *KIT*, and *PIK3CA* in <10% of the cases. For comparison of pulmonary versus extra-pulmonary NEC, Bergsland et al. [33] conducted genomic analysis of ~600 SCLC cases and 270 poorly differentiated GEP-NEC cases, and showed that *TP53* and *RB1* mutations were prominent in SCLC (90% and 67%, respectively), *MEN1* and *DAXX* mutations were frequent in pancreatic NEC (33% and 20%, respectively), and *APC* and *KRAS* mutations were often noted in colon NEC (47% and 37%, respectively. Such heterogeneity requires different treatment approaches for NEC tumors in different primary organs.

In terms of the fusion genes, our analysis identified *FGFR3* rearrangement (*FGFR3*-*TACC3*) in one case. *FGFR3*-*TACC3* is oncogenic, as it induces mitotic and chromosomal-segregation defects and triggers aneuploidy [34]. According to previous studies, *FGFR3*-*TACC3* is detected in 3% of glioblastoma cases and 0.5% of lung adenocarcinoma cases [34, 35]. Helsten et al. [36] reported that 3.7% of 107 NEN cases harbored *FGFR1* amplification, whereas *FGFR3* mutations or rearrangements were not found. There are no data concerning the incidence of this fusion gene in poorly differentiated NEC. Currently, erdafitinib and pemigatinib are approved for use in the United States for metastatic urothelial carcinoma with *FGFR2* or *FGFR3* aberrations and for cholangiocarcinoma with an *FGFR2* rearrangement, respectively [37, 38]. In the present study, the results suggested that erdafitinib might be a promising option for some metastatic cases of NEC of the head and neck.

Another important issue is the clinical validity of immunotherapy for head and neck NEC. In the United States, the immune checkpoint inhibitor (ICI) pembrolizumab has been approved for patients with unresectable or metastatic solid tumors with high TMB [39]. The FoundationOne CDx assay was adopted as the companion diagnostic, for which TMB-high was defined as ≥10 mut/Mb, and our three patients with a TMB ≥ 10 mut/Mb might be suitable candidates for pembrolizumab treatment in the future. Along with the TMB, MSI status is another important factor for estimating a good response to ICI. None of our patients exhibited an MSI quantitative score ≥0.1, which is considered MSI-stable by PCR fragment analysis [40]. As Vanderwalde et al. [41] reported, discordance between the TMB and MSI status is often observed, and the rate varies among different types of malignancy. The Brinkman index in our three patients with high TMB was >200, and a relationship with tobacco consumption was suspected. A more comprehensive assessment is required to determine the clinical application of ICIs. Clinical data on the utility of ICIs for NEC are limited. A phase II study of pembrolizumab for SCC of the lower genital tract and a phase II study of avelumab for extrapulmonary NEC according to the 2010 WHO classification exhibited a progression-free rate of 0% at 27 weeks and a disease-control rate of 32% at 8 weeks, respectively [42, 43]. According to the joint analysis of twophase II studies of pembrolizumab for extrapulmonary NEC (2010 WHO classification) that enrolled 29 patients, the overall response rate was 3% and disease control-rate at 18 weeks was 10% [44]. Further investigations, including studies of the effects of combinations with cytotoxic agents, are essential to enhance the clinical efficacy of ICIs. Briefly, limited data concerning NEC derived from other sites have not shown a strong role for immunotherapy, and the role of high TMB in clinical application of ICIs remains unclear.

This study had some limitations. First, although this is the first genomic analysis of an extremely rare disease, the sample size was small. Furthermore, because of the high proportion of biopsied samples, a considerable number of specimens were minute and unsuitable for genomic analysis. In relation to this point, a biopsied lesion is only a part of the entire tumor, and it should be noted that pure NEC morphology on biopsy does not completely deny the coexistence of other histological components. Second, we used targeted sequencing but not whole-exome or whole-genome sequencing. Although the number of genes (>500) in this study was generally satisfactory for identifying druggable cases, it was insufficient to reveal the whole picture of the genomic background in head and neck NEC. In addition, normal reference samples were not sequenced, and a complete distinction of single-nucleotide polymorphisms was difficult. Third, cell lines derived from NEC of the head and neck are not available commercially; therefore, in vitro and in vivo functional analyses of the detected mutations could not be conducted. Finally, precision-medicine approaches are generally preferred for unresectable or metastatic cases, but it should be noted that the genetic information obtained in this study did not directly present novel therapeutic options in most of our patients.

In conclusion, as the first comprehensive analysis of head and neck NEC, we revealed the clinicopathological and genomic features of this rare disease. Clinically, patients with locally advanced disease had favorable outcomes, although the pathological features were aggressive. Targeted-capture sequencing of 523 cancer-related genes revealed lower prevalence of mutations in *TP53* and *RB1* as compared with that in SCLC, as well as detected *FGFR3*-*TACC3* gene fusion and deleterious/likely deleterious gene variants in *NOTCH1* and several genes encoding components of the PI3K/AKT/mTOR pathway.

## Supplementary information

Supplementary Table 1

## Data Availability

The datasets used and/or analyzed during this study are available from the corresponding author upon reasonable request.
